# Supplementary material for: Diplodia seriata Isolated from Declining Olive Trees in Salento (Apulia, Italy): Pathogenicity Trials Give a Glimpse That It Is More Virulent to Drought-Stressed Olive Trees and in a Warmth-Conditioned Environment
Source: Plants (Basel). 2024 Aug 13;13(16):2245. doi: 10.3390/plants13162245 (PMC11358911; doi:10.3390/plants13162245)
Supplement: Supplementary file 1 [file plants-13-02245-s001.zip › Figure_S1.pdf]

Title:

Diplodia seriata isolated from declining olive trees in Salento (Apulia, Italy): pathogenicity trials give a glimpse that it is virulent to drought-stressed olive trees in a warmth-conditioned environment

Journal name:

Plants MDPI

Authors:

Giuliano Manetti, Lorenzo Sciarroni, Angela Brunetti, Valentina Lumia, Sara Bechini, Paolo Marangi, Massimo Reverberi, Marco Scortichini

and Massimo Pilotti

Corresponding author:

Massimo Pilotti

Affiliation:

Institution: Council for Agricultural Research and Economics; Department: Research Center for Plant Protection and Certification (CREA-DC) - Via C. G. Bertero 22, 00156 Rome, Italy

E-mail address:

massimo.pilotti@crea.gov.it

Figure S1. Phylogenetic tree of *Diplodia* species, based on ITS + TEF1-α + TUB2 data set and including the botryosphaeriaceous isolates from Salento (Apulia, Italy): CREA-DC TPR OL.437, 464, 548, 700 (shaded in blue-sky). The evolutionary history was inferred by using the Maximum Likelihood method and General Time Reversible model (Nei and Kumar 2000). The tree with the highest log likelihood (-5364.13) is shown. The percentage of trees in which the associated taxa clustered together (bootstrap support value) is shown next to the branches only for values higher than 49%. Initial tree(s) for the heuristic search were obtained automatically by applying Neighbor-Join and BioNJ algorithms to a matrix of pairwise distances estimated using the Maximum Composite Likelihood (MCL) approach, and then selecting the topology with superior log likelihood value. The tree is drawn to scale, with branch lengths measured in the number of substitutions per site. This analysis involved 85 nucleotide sequences. There were a total of 1333 positions in the final dataset. Evolutionary analyses were conducted in MEGA X (Kumar et al. 2018).

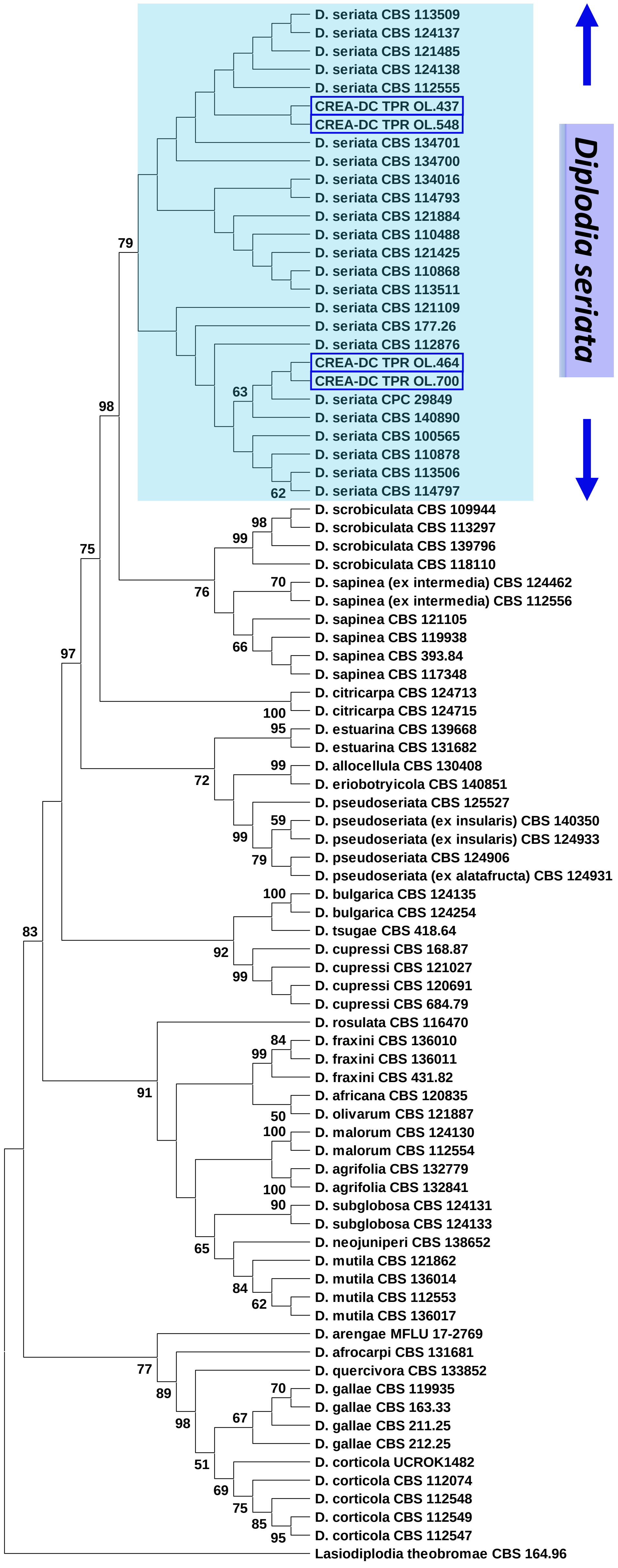
D. seriata CBS 113509
D. seriata CBS 124137
D. seriata CBS 121485
D. seriata CBS 124138
D. seriata CBS 112555
CREA-DC TPR OL.437
CREA-DC TPR OL.548
D. seriata CBS 134701
D. seriata CBS 134700
D. seriata CBS 134016
D. seriata CBS 114793
D. seriata CBS 121884
D. seriata CBS 110488
D. seriata CBS 121425
D. seriata CBS 110868
D. seriata CBS 113511
D. seriata CBS 121109
D. seriata CBS 177.26
D. seriata CBS 112876
CREA-DC TPR OL.464
CREA-DC TPR OL.700
D. seriata CPC 29849
D. seriata CBS 140890
D. seriata CBS 100565
D. seriata CBS 110878
D. seriata CBS 113506
D. seriata CBS 114797
Diplodia seriata
D. scrobiculata CBS 109944
D. scrobiculata CBS 113297
D. scrobiculata CBS 139796
D. scrobiculata CBS 118110
D. sapinea (ex intermedia) CBS 124462
D. sapinea (ex intermedia) CBS 112556
D. sapinea CBS 121105
D. sapinea CBS 119938
D. sapinea CBS 393.84
D. sapinea CBS 117348
D. citricarpa CBS 124713
D. citricarpa CBS 124715
D. estuarina CBS 139668
D. estuarina CBS 131682
D. allocellula CBS 130408
D. eriobotryicola CBS 140851
D. pseudoseriata CBS 125527
D. pseudoseriata (ex insularis) CBS 140350
D. pseudoseriata (ex insularis) CBS 124933
D. pseudoseriata CBS 124906
D. pseudoseriata (ex alatafructa) CBS 124931
D. bulgarica CBS 124135
D. bulgarica CBS 124254
D. tsugae CBS 418.64
D. cupressi CBS 168.87
D. cupressi CBS 121027
D. cupressi CBS 120691
D. cupressi CBS 684.79
D. rosulata CBS 116470
D. fraxini CBS 136010
D. fraxini CBS 136011
D. fraxini CBS 431.82
D. africana CBS 120835
D. olivarum CBS 121887
D. malorum CBS 124130
D. malorum CBS 112554
D. agrifolia CBS 132779
D. agrifolia CBS 132841
D. subglobosa CBS 124131
D. subglobosa CBS 124133
D. neojuniperi CBS 138652
D. mutila CBS 121862
D. mutila CBS 136014
D. mutila CBS 112553
D. mutila CBS 136017
D. arengae MFLU 17-2769
D. afrocarpi CBS 131681
D. quercivora CBS 133852
D. gallae CBS 119935
D. gallae CBS 163.33
D. gallae CBS 211.25
D. gallae CBS 212.25
D. corticola UCROK1482
D. corticola CBS 112074
D. corticola CBS 112548
D. corticola CBS 112549
D. corticola CBS 112547
Lasiodiplodia theobromae CBS 164.96
