# Supplementary material for: Diplodia seriata Isolated from Declining Olive Trees in Salento (Apulia, Italy): Pathogenicity Trials Give a Glimpse That It Is More Virulent to Drought-Stressed Olive Trees and in a Warmth-Conditioned Environment
Source: Plants (Basel). 2024 Aug 13;13(16):2245. doi: 10.3390/plants13162245 (PMC11358911; doi:10.3390/plants13162245)
Supplement: Supplementary file 1 [file plants-13-02245-s001.zip › Text_Table_S1.pdf]

Title:

Diplodia seriata isolated from declining olive trees in Salento (Apulia, Italy): pathogenicity trials give a glimpse that it is virulent to drought-stressed olive trees in a warmth-conditioned environment

Journal name:

Plants MDPI

Authors:

Giuliano Manetti, Lorenzo Sciarroni, Angela Brunetti, Valentina Lumia, Sara Bechini, Paolo Marangi, Massimo Reverberi, Marco Scortichini and Massimo Pilotti

Corresponding author:

Massimo Pilotti

Affiliation:

Institution: Council for Agricultural Research and Economics; Department: Research Center for Plant Protection and Certification (CREA-DC) - Via C. G. Bertero 22, 00156 Rome, Italy

E-mail address:

massimo.pilotti@crea.gov.it

Text Table S1. Methods for DNA extraction, PCR amplifications and sequencing of DNA regions of *Diplodia seriata* isolated from olive trees in Salento (Apulia, Italy). In Table A the DNA regions amplified and primers used are reported. In Table B the thermal cycling for each DNA region is described. Below sequences generated in this study and their accession numbers are listed.

Genomic DNA (gDNA) of fungal isolates was obtained from 100 mg mycelium sampled from axenic cultures actively growing on sterile cellophane discs (BIO-RAD, Hercules, CA, USA) layered on PDA plates and collected by scraping with a sterile pipette tip. Mycelium samples were powdered with liquid nitrogen, then gDNA was extracted using a DNeasy Plant Mini Kit (Qiagen, Hilden, Germany) following the manufacturer's instructions.

PCR amplifications were carried out on a PCR T100™ Thermal Cycler (BIO-RAD). Reaction assembly was in 50μl volume and contained each primer at 0.2 μM, 2.5 U of High Fidelity Platinum Taq DNA Polymerase (Invitrogen, Thermo Fisher Scientific, Waltham, MA, USA), 0.2 mM (ITS) or 0.4 mM (TUB2 e TEF1-α) of each dNTP, in the buffer supplied by the Taq polymerase manufacturer.

Primer sequences and citations are reported in table A. Thermal cycling is reported in table B.

The PCR products were analysed by agarose gel electrophoresis, excised from gel, and eluted with ISOLATE II PCR and gel kit (Meridian Bioscience, River Hills Drive, Cincinnati, OH, USA) following the manufacturer's instructions. Then amplicons were directly sequenced in both directions by Sanger technology (Bio-Fab research s.r.l. Rome Italy).

Table A. DNA regions amplified and primers used

DNA region	Forward primer	Sequence	Reverse primer	Sequence	Citation
ITS	ITS5	5′-GGAAGTAAAAGTCGTAACAAGG-3′	ITS4	5′-TCCTCCGCTTATTGATATGC-3′	[93]
TUB2	T1-β tubulin[1]	5′-AACATGCGTGAGATTGTAAGT-3′	Bt2b[2]	5′-ACCCTCAGTGTAGTGACCCTTGGC-3′	[1] [94]<br>[2] [95]
TEF1-α	EF688F	5′-CGGTCACTTGATCTACAAGTG C-3′	EF1251R	5′-CCTCGAACTCACCAGTACCG-3′	[96]

Table B. Thermal cycling (35 cycles) to amplify DNA regions used for fungal identification (Blast and phylogeny)

Loci DNA	Initial denaturation	Denaturation for each cycle	Annealing	Extension	Final extension
ITS	94°C, 3min	94°C, 30s	50°C, 30s	68°C, 1min 15s	68°C, 10min
TEF1-α	94°C, 3min	94°C, 30s	55°C, 45s	68°C, 1min 30s	68°C, 10min
TUB2	94°C, 3min	94°C, 30s	50°C, 30s	68°C, 1min	68°C, 10min

References

93. White, T.; Bruns, T.; Lee, S.; Taylor, J.; Innis, M.; Gelfand, D.; Sninsky, J. Amplification and Direct Sequencing of Fungal Ribosomal RNA Genes for Phylogenetics. In *Pcr Protocols: a Guide to Methods and Applications,*; 1990; Vol. 31, pp. 315–322.
94. Glass, N.L.; Donaldson, G.C. Development of Primer Sets Designed for Use with the PCR to Amplify Conserved Genes from Filamentous Ascomycetes. *Appl Environ Microbiol* **1995**, *61*, 1323–1330, doi:10.1128/aem.61.4.1323-1330.1995.
95. O'Donnell, K.; Cigelnik, E. Two Divergent Intragenomic rDNA ITS2 Types within a Monophyletic Lineage of the Fungus*Fusarium*Are Nonorthologous. *Molecular Phylogenetics and Evolution* **1997**, *7*, 103–116, doi:10.1006/mpev.1996.0376.
96. Alves, A.; Crous, P.; Correia, A.; Phillips, A. Morphological and Molecular Data Reveal Cryptic Species in Lasiodiplodia Theobromae. *Fungal Diversity 28 (2008)* **2008**, *28*.

Sequences

>accession Nos_fungal isolate code DNA locus:

ITS

>PP712106_CREA-DC TPR OL.437 ITS
TTTCCGTAGGTGAACCTGCGGAAGGATCATTACCGAGTTCTCGGGCTTCGGCTCGAATCTCCCACCCTTTGTG
AACATACCTCTGTTGCTTTGGCGGCTCTTTGCCGCGAGGAGGCCCTCGCGGGCCCCCCCGCGCGCTTTCTGCC
AGAGGACCTTCAAACTCCAGTCAGTAAACGTCGACGTCTGATAAACAAGTTAATAAACTAAAACTTTCAACAA
CGGATCTCTTGGTTCTGGCATCGATGAAGAACGCAGCGAAATGCGATAAGTAATGTGAATTGCAGAATTCAGT
GAATCATCGAATCTTTGAACGCACATTGCGCCCCCTGGCATTCCGGGGGGCATGCCTGTTCGAGCGTCATTAC
AACCCTCAAGCTCTGCTTGGTATTGGGCGCCGTCCTCTCTGCGGACGCGCCTTAAAGACCTCGGCGGTGGCTG
TTCAGCCCTCAAGCGTAGTAGAATACACCTCGCTTTGGAGCGGTTGGCGTCGCCCGCCGGACGAACCTTCTGA
ACTTTTCTCAAGGTTGACCTCGGATCAGGTAGGGATACCCGCTGAACTTAA

>PP712107_CREA-DC TPR OL.464 ITS
TTTCCGTAGGTGAACCTGCGGAAGGATCATTACCGAGTTCTCGGGCTTCGGCTCGAATCTCCCACCCTTTGTG
AACATACCTCTGTTGCTTTGGCGGCTCTTTGCCGCGAGGAGGCCCTCGCGGGCCCCCCCGCGCGCTTTCTGCC
AGAGGACCTTCAAACTCCAGTCAGTAAACGTCGACGTCTGATAAACAAGTTAATAAACTAAAACTTTCAACAA
CGGATCTCTTGGTTCTGGCATCGATGAAGAACGCAGCGAAATGCGATAAGTAATGTGAATTGCAGAATTCAGT
GAATCATCGAATCTTTGAACGCACATTGCGCCCCCTGGCATTCCGGGGGGCATGCCTGTTCGAGCGTCATTAC
AACCCTCAAGCTCTGCTTGGTATTGGGCGCCGTCCTCTCTGCGGACGCGCCTTAAAGACCTCGGCGGTGGCTG
TTCAGCCCTCAAGCGTAGTAGAATACACCTCGCTTTGGAGCGGTTGGCGTCGCCCGCCGGACGAACCTTCTGA
ACTTTTCTCAAGGTTGACCTCGGATCAGGTAGGGATACCCGCTGAACTTAA

>PP712108_CREA-DC TPR OL.548 ITS
TTTCCGTAGGTGAACCTGCGGAAGGATCATTACCGAGTTCTCGGGCTTCGGCTCGAATCTCCCACCCTTTGTG
AACATACCTCTGTTGCTTTGGCGGCTCTTTGCCGCGAGGAGGCCCTCGCGGGCCCCCCCGCGCGCTTTCTGCC
AGAGGACCTTCAAACTCCAGTCAGTAAACGTCGACGTCTGATAAACAAGTTAATAAACTAAAACTTTCAACAA
CGGATCTCTTGGTTCTGGCATCGATGAAGAACGCAGCGAAATGCGATAAGTAATGTGAATTGCAGAATTCAGT
GAATCATCGAATCTTTGAACGCACATTGCGCCCCCTGGCATTCCGGGGGGCATGCCTGTTCGAGCGTCATTAC
AACCCTCAAGCTCTGCTTGGTATTGGGCGCCGTCCTCTCTGCGGACGCGCCTTAAAGACCTCGGCGGTGGCTG
TTCAGCCCTCAAGCGTAGTAGAATACACCTCGCTTTGGAGCGGTTGGCGTCGCCCGCCGGACGAACCTTCTGA
ACTTTTCTCAAGGTTGACCTCGGATCAGGTAGGGATACCCGCTGAACTTAA

>PP712109_CREA-DC TPR OL.700 ITS
TTTCCGTAGGTGAACCTGCGGAAGGATCATTACCGAGTTCTCGGGCTTCGGCTCGAATCTCCCACCCTTTGTG
AACATACCTCTGTTGCTTTGGCGGCTCTTTGCCGCGAGGAGGCCCTCGCGGGCCCCCCCGCGCGCTTTCTGCC
AGAGGACCTTCAAACTCCAGTCAGTAAACGTCGACGTCTGATAAACAAGTTAATAAACTAAAACTTTCAACAA
CGGATCTCTTGGTTCTGGCATCGATGAAGAACGCAGCGAAATGCGATAAGTAATGTGAATTGCAGAATTCAGT
GAATCATCGAATCTTTGAACGCACATTGCGCCCCCTGGCATTCCGGGGGGCATGCCTGTTCGAGCGTCATTAC
AACCCTCAAGCTCTGCTTGGTATTGGGCGCCGTCCTCTCTGCGGACGCGCCTTAAAGACCTCGGCGGTGGCTG
TTCAGCCCTCAAGCGTAGTAGAATACACCTCGCTTTGGAGCGGTTGGCGTCGCCCGCCGGACGAACCTTCTGA
ACTTTTCTCAAGGTTGACCTCGGATCAGGTAGGGATACCCGCTGAACTTAA

beta-tubulin 2(TUB2)

>PP727264__CREA-DC TPR OL.437 TUB2
TTCTGCGGCAGCCCGACTGTTTTTGACGCGTCCCCTGACAGCCCCGCGTCTTTGCCCCCGGCCGCAACAACAG
GCCAACCATTGCTAACATCGCTTTTTCGCACCCATAGGTTCACCTTCAGACCGGCCAATGCGTAAGTCTCTTC
CCGCCTGCTGTGTTCGCTGCATCGCGCTGACTTTTCCCAGGGTAACCAAATTGGTGCTGCCTTCTGGTTTGTT
GCCAAAACACTCCCGCTGCCGCGCCCCCCGCTGACGCCAATCGACACCACAGGCAGACTATCTCTGGCGAGCA
CGGCCTGGACGGCTCCGGCGTGTAAGTTTGCGCTGTCTTTGCCGCGCTCTGCAATCGCTGACCCTTGGCAGCT
ACAATGGCACCTCCGACCTCCAGCTGGAGCGCATGAACGTCTACTTCAACGAGGTACTCTCTACTAGTTAGAC
AAACACGTAAAGTATGGCAATCTTCTGAACGCGCAGCAGGCATCCAACAATAAGTACGTTCCTCGTGCTGTCC
TCGTTGACCTCGAGCCCGGCACCATGGATGCCGTCCGCGCCGGCCCCTTCGGCCAGCTCTTCCGTCCCGACAA
CTTCGTTTTCGGCCAGTCTGGTGCCGGTAACAACTGG

>PP727265_CREA-DC TPR OL.464 TUB2
TTCTGCGGCAGCCCGACTGTTTTTGACGCGTCCCCTGACAGCCCCGCGTCTTTGCCCCCGGCCGCAACAACAG
GCCAACCATTGCTAACATCGCTTTTTCGCACCCATAGGTTCACCTTCAGACCGGCCAATGCGTAAGTCTCTTC
CCGCCTGCTGTGTTCGCTGCATCGCGCTGACTTTTCCCAGGGTAACCAAATTGGTGCTGCCTTCTGGTTTGTT
GCCAAAACACTCCCGCTGCCGCGCCCCCCGCTGACGCCAATCGACACCACAGGCAGACTATCTCTGGCGAGCA
CGGCCTGGACGGCTCCGGCGTGTAAGTTTGCGCTGTCTTTGCCGCGCTCTGCAATCGCTGACCCTTGGCAGCT
ACAATGGCACCTCCGACCTCCAGCTGGAGCGCATGAACGTCTACTTCAACGAGGTACTCTCTACTAGTTAGAC
AAACACGTAAAGTATGGCAATCTTCTGAACGCGCAGCAGGCATCCAACAATAAGTACGTTCCTCGTGCTGTCC
TCGTTGACCTCGAGCCCGGCACCATGGATGCCGTCCGCGCCGGCCCCTTCGGCCAGCTCTTCCGTCCCGACAA
CTTCGTTTTCGGCCAGTCTGGTGCCGGTAACAACTGG

>PP727266_CREA-DC TPR OL.548 TUB2
TTCTGCGGCAGCCCGACTGTTTTTGACGCGTCCCCTGACAGCCCCGCGTCTTTGCCCCCGGCCGCAACAACAG
GCCAACCATTGCTAACATCGCTTTTTCGCACCCATAGGTTCACCTTCAGACCGGCCAATGCGTAAGTCTCTTC
CCGCCTGCTGTGTTCGCTGCATCGCGCTGACTTTTCCCAGGGTAACCAAATTGGTGCTGCCTTCTGGTTTGTT
GCCAAAACACTCCCGCTGCCGCGCCCCCCGCTGACGCCAATCGACACCACAGGCAGACTATCTCTGGCGAGCA
CGGCCTGGACGGCTCCGGCGTGTAAGTTTGCGCTGTCTTTGCCGCGCTCTGCAATCGCTGACCCTTGGCAGCT
ACAATGGCACCTCCGACCTCCAGCTGGAGCGCATGAACGTCTACTTCAACGAGGTACTCTCTACTAGTTAGAC
AAACACGTAAAGTATGGCAATCTTCTGAACGCGCAGCAGGCATCCAACAATAAGTACGTTCCTCGTGCTGTCC
TCGTTGACCTCGAGCCCGGCACCATGGATGCCGTCCGCGCCGGCCCCTTCGGCCAGCTCTTCCGTCCCGACAA
CTTCGTTTTCGGCCAGTCTGGTGCCGGTAACAACTGG

>PP727267_CREA-DC TPR OL.700 TUB2
TTCTGCGGCAGCCCGACTGTTTTTGACGCGTCCCCTGACAGCCCCGCGTCTTTGCCCCCGGCCGCAACAACAG
GCCAACCATTGCTAACATCGCTTTTTCGCACCCATAGGTTCACCTTCAGACCGGCCAATGCGTAAGTCTCTTC
CCGCCTGCTGTGTTCGCTGCATCGCGCTGACTTTTCCCAGGGTAACCAAATTGGTGCTGCCTTCTGGTTTGTT
GCCAAAACACTCCCGCTGCCGCGCCCCCCGCTGACGCCAATCGACACCACAGGCAGACTATCTCTGGCGAGCA
CGGCCTGGACGGCTCCGGCGTGTAAGTTTGCGCTGTCTTTGCCGCGCTCTGCAATCGCTGACCCTTGGCAGCT
ACAATGGCACCTCCGACCTCCAGCTGGAGCGCATGAACGTCTACTTCAACGAGGTACTCTCTACTAGTTAGAC
AAACACGTAAAGTATGGCAATCTTCTGAACGCGCAGCAGGCATCCAACAATAAGTACGTTCCTCGTGCTGTCC
TCGTTGACCTCGAGCCCGGCACCATGGATGCCGTCCGCGCCGGCCCCTTCGGCCAGCTCTTCCGTCCCGACAA
CTTCGTTTTCGGCCAGTCTGGTGCCGGTAACAACTGG

Translation elongation factor 1-alpha (TEF1-alpha)

>PP727268_CREA-DC TPR OL.437 TEF1-alpha
GGTGGTATTGACAAGCGTACCATCGAGAAGTTCGAGAAGGTAAGAGGATTTTTCCCGCTCCCGCACTGCGTGC
ACAGCCCACCTTATCGCTCGGTGAGGGGCATTTTTCGTGGTGGGGTTTGGCCCGCGCTAAACCTCGTCTGAGC
TCGGCAAACTGGCCGCACTTGGTTTTTTGCGACCGGCGTCTGGCCGATGCGCCCCTCACTAGCGAGCAATGCC
CCGACCACTCATGTGCTCTCGTACGACCACAGGCTAACGCACGCCACAACAGGAAGCCGCCGAGCTCGGCAAG
GGTTCCTTCAAGTACGCCTGGGTCCTTGACAAGCTCAAGGCCGAGCGTGAGCGTGGTATCACCATCGACATCG
CCCTCTGGAAGTTCGAGACCCCGAAGTACTATGTCACCGTCATTGACGCCCCCGGTCACCGTGACTTCATCAA
GAACATGATCACTGGTACCTCGCAGGCCGACTGCGCCATTCTCATCATTGCCGC

>PP727269_CREA-DC TPR OL.464 TEF1-alpha
GGTGGTATTGACAAGCGTACCATCGAGAAGTTCGAGAAGGTAAGAGGATTTTTCCTGCTCCCGCACCGCGTGC
ACAGCCCACCTTATCGCTCGGTGAGGGGCATTTTTCGTGGTGGGGTTTGGCCCGCGCTAAACCTCGTCTGAGC
TCGGCAAACTGGCCGCACTTGGTTTTTTGCGACCGGCGTCAGGCCGATGCGCCCCTCACTAGCGAGCAATGCC
CCGACCACTCATGTGCTCTCGTGCGACCACAGGCTAACGCACGCCACAACAGGAAGCCGCCGAGCTCGGCAAG
GGTTCCTTCAAGTACGCCTGGGTCCTTGACAAGCTTAAGGCCGAGCGTGAGCGTGGTATCACCATCGACATCG
CCCTCTGGAAGTTCGAGACCCCGAAGTACTATGTCACCGTCATTGACGCCCCCGGTCACCGTGACTTCATCAA
GAACATGATCACTGGTACCTCGCAGGCCGACTGCGCCATTCTCATCATTGCCGC

>PP727270_CREA-DC TPR OL.548 TEF1-alpha
GGTGGTATTGACAAGCGTACCATCGAGAAGTTCGAGAAGGTAAGAGGATTTTTCCCGCTCCCGCACTGCGTGC
ACAGCCCACCTTATCGCTCGGTGAGGGGCATTTTTCGTGGTGGGGTTTGGCCCGCGCTAAACCTCGTCTGAGC
TCGGCAAACTGGCCGCACTTGGTTTTTTGCGACCGGCGTCTGGCCGATGCGCCCCTCACTAGCGAGCAATGCC
CCGACCACTCATGTGCTCTCGTACGACCACAGGCTAACGCACGCCACAACAGGAAGCCGCCGAGCTCGGCAAG
GGTTCCTTCAAGTACGCCTGGGTCCTTGACAAGCTCAAGGCCGAGCGTGAGCGTGGTATCACCATCGACATCG
CCCTCTGGAAGTTCGAGACCCCGAAGTACTATGTCACCGTCATTGACGCCCCCGGTCACCGTGACTTCATCAA
GAACATGATCACTGGTACCTCGCAGGCCGACTGCGCCATTCTCATCATTGCCGC

>PP727271_CREA-DC TPR OL.700 TEF1-alpha
GGTGGTATTGACAAGCGTACCATCGAGAAGTTCGAGAAGGTAAGAGGATTTTTCCTGCTCCCGCACCGCGTGC
ACAGCCCACCTTATCGCTCGGTGAGGGGCATTTTTCGTGGTGGGGTTTGGCCCGCGCTAAACCTCGTCTGAGC
TCGGCAAACTGGCCGCACTTGGTTTTTTGCGACCGGCGTCAGGCCGATGCGCCCCTCACTAGCGAGCAATGCC
CCGACCACTCATGTGCTCTCGTGCGACCACAGGCTAACGCACGCCACAACAGGAAGCCGCCGAGCTCGGCAAG
GGTTCCTTCAAGTACGCCTGGGTCCTTGACAAGCTTAAGGCCGAGCGTGAGCGTGGTATCACCATCGACATCG
CCCTCTGGAAGTTCGAGACCCCGAAGTACTATGTCACCGTCATTGACGCCCCCGGTCACCGTGACTTCATCAA
GAACATGATCACTGGTACCTCGCAGGCCGACTGCGCCATTCTCATCATTGCCGC
